# Epistatic chromatin remodeling during barley response to powdery mildew by ATAC-Seq

**DOI:** 10.1038/s41597-026-07313-0

**Published:** 2026-05-08

**Authors:** Valeria Velásquez-Zapata, Schuyler D. Smith, Gregory Fuerst, Roger P. Wise

**Affiliations:** 1 Department of Plant Pathology, Entomology, and Microbiology, Iowa State University, Ames, IA 50011 USA; 2 Oak Ridge Institute for Science and Education (ORISE), Oak Ridge, TN 37831 USA; 3 USDA-Agricultural Research Service, Corn Insects and Crop Genetics Research Unit, Iowa State University, Ames, IA 50011 USA

**Keywords:** Gene expression, Plant genetics, Epigenetics analysis

## Abstract

Understanding the molecular basis of plant-pathogen interactions is critical for advancing crop protection strategies. Powdery mildew, caused by the obligate fungal pathogen *Blumeria hordei* (*Bh*), is a threat to barley production worldwide. We exploited time-course ATAC-Seq of barley and derived immune mutants infected with *Bh* to infer chromatin accessibility influenced by the genetic interactions of *mildew locus a6* (*Mla6)*, encoding a nucleotide binding leucine-rich repeat (NLR) immune receptor, and *Blufensin1* (*Bln1)*, a basal defense regulator. Sampling at 0, 16, 20, and 32 hours after inoculation captured key pathogen developmental stages representing fungal penetration and haustorial development, respectively. Validation of the dataset was accomplished by calculating general ATAC-Seq peak metrics and comparison with paired RNA-Seq data. ATAC-Seq and RNA-Seq results were correlated, highlighting in particular chromatin-mediated epistatic interactions, demonstrating that the dataset could provide insight into regulatory chromatin architecture. These results offer a valuable dataset for dissecting transcriptional networks involved in barley immune responses.

## Background & Summary

Investigating the intricate molecular mechanisms governing plant-pathogen interactions is pivotal for designing effective strategies to enhance food security^1^. Powdery mildew fungi are a common class of pathogens that infect thousands of plants, including cereal crops such as barley and wheat, causing substantial yield loss^2^. The establishment of specialized high-throughput sequencing technologies, including ATAC-Seq and RNA-Seq, has revolutionized the ability to dissect complex biological systems and identify key genetic factors influencing disease resistance^3^. These omics approaches have not only deepened insights into plant immunity but have also shed light on chromatin-level regulatory mechanisms, such as histone acetylation and methylation, which play critical roles in modulating gene expression during pathogen infection^4^.

Assay for transposase-accessible chromatin using sequencing (ATAC-Seq) is a technique used to map chromatin accessibility across the genome, thereby revealing regulatory regions and their dynamic changes in response to diverse stimuli. Previous omic studies in barley infected with powdery mildew, caused by the ascomycete fungus, *Blumeria hordei (Bh)*, revealed a large transcriptional network in response to pathogen attack^5–7^. Expressed genes often exhibit an open chromatin configuration, suggesting the presence of chromatin modifications in the promoter region of transcription factors and genes associated with plant immune response, such as salicylic acid- and pattern-triggered immunity (PTI)-responsive genes^8^. To investigate chromatin accessibility in barley during powdery mildew infection, ATAC-Seq can be used to assay resistant and susceptible leaf tissues at several timepoints post-inoculation. This approach allows for the identification of genomic regions exhibiting altered accessibility, potentially indicating the involvement of regulatory elements involved in pathogen recognition, signaling, and defense responses. The resulting data can be analyzed to identify regions of increased or decreased chromatin accessibility, providing insights into the activation/repression of specific genes, or larger gene regions. Analysis of ATAC-Seq data could show altered accessibility within promoter regions of defense-related genes, signifying enhanced or reduced transcriptional activity. Changes in chromatin accessibility may also occur in distal regulatory elements, such as enhancers and silencers, modulating gene expression patterns.

To characterize chromatin accessibility of barley during powdery mildew infection, a set of isogenic mutant genotypes and timepoints, as described by Velásquez-Zapata and colleagues^6^, were subjected to ATAC sequencing. We designed these studies around two diverse genes in host immunity, *Mildew locus a6* (*Mla6), a* nucleotide binding leucine-rich repeat (NLR) immune receptor, and the *Blufensin1* (*Bln1)* basal defense regulator, which influence immunity in a resistance (*R*)-gene dependent and *R*-gene independent manner, respectively. In addition to the CI 16151 resistant progenitor (*Mla6, Bln1*), three fast-neutron derived mutants were utilized: the susceptible *mla6*-m18982, the resistant *bln1*-m19089, and the susceptible double mutant (*mla6 + bln1*)-m19028^6,9^. Four infection timepoints (0, 16, 20, 32 HAI) were taken to sample across key pathogen life cycle events, for example, conidial germination, appressorial penetration and haustorial development. Validation of the ATAC-Seq dataset was performed by calculating general metrics such as peak distribution and classification. In addition, we checked for correlation with gene expression profiles from previous RNA-Seq data by checking the presence of epistatic events at the chromatin level^6^.

## Methods

### Plant materials and tissue collection

The wild-type progenitor, CI 16151 (*Mla6, Bln1*), was developed by the introgression of the *Mla6* nucleotide binding leucine rich repeat (NLR) immune receptor into the Manchuria background^10^ and maintained by single seed descent. CI 16151 derived mutants [m18982 (*mla6, Bln1*); m19089 (*Mla6, bln1*); m19028 (*mla6, bln1*)] were generated via fast-neutrons (4 Gy Nf)^11,12^ and further characterized as described by^6,9^.

Seven-day old barley (*Hordeum vulgare*) first leaves were grown in 20 × 30-cm trays using sterilized potting mix (Sunshine Mix #1, Sungrow) in a climate-controlled greenhouse (16 h supplemental light, 20–22 C). Trays were blocked by biological replicate and plant genotype with seedling rows randomly assigned to one of four harvest timepoints (0, 16, 20, and 32 HAI). First leaf seedlings were inoculated with a high density (84 ± 19 spores/mm^2^) of *Blumeria hordei* (*Bh)* isolate 5874 (*AVR*_*a6*_) and immediately transferred to a growth chamber (16 h light / 8 dark, 18 C)^5^. One gram of pooled tissue was collected for each timepoint and flash frozen in liquid nitrogen. Four biological replicates were collected for each genotype / timepoint combination and processed for ATAC-Seq protocols.

### ATAC-Seq protocol

ATAC-Seq libraries were prepared by REquest Genomics (Athens, GA, USA). Barley tissue was chopped on ice using a razor blade for ~2 minutes in 600 μL of pre-chilled Nuclei Isolation Buffer (NIB)^13^. The chopped tissue was filtered across a 40-μm cell strainer and subjected to centrifugation at 500 rcf for 5 minutes at 4 C. The supernatant was removed, and the pellet was resuspended in 500 μL of NIB. Samples were then put through a 20-μm cell strainer and loaded onto the surface of 1 mL of 35% Percoll buffer, which was prepared by mixing 35% Percoll with 65% NIB wash buffer. The nuclei were centrifuged at 500 rcf for 10 minutes at 4 C. The supernatant was gently withdrawn, and pellets were washed in 100 μL of 1X TAPS buffer (5X TAPS buffer: 50 mM TAPS-NaOH, pH8.0; 25 mM MgCl2)^14^. Subsequently, the nuclei were reconstituted in 30 μL of 2.5X TAPS buffer. A total of 5 μL of nuclei were diluted tenfold and stained with DAPI (Sigma Cat. D9542) to evaluate the condition and abundance using a hemocytometer. A total of 50,000 nuclei were used per library preparation. The Tn5 assembly and library preparation procedures were conducted following previously established methods^14^. Libraries were sequenced by Active Motif (Carlsbad, CA, USA) on an Illumina NovaSeq 6000 using paired-end 150 bp reads. The run configuration included 150 bp for Read 1 (R1), 8 bp for Index 1 (I1), 8 bp for Index 2 (I2), and 150 bp for Read 2 (R2).

### ATAC-Seq read processing

The nf-core/atacseq pipeline (https://nf-co.re/atacseq/2.1.2/) was used to process ATAC-Seq data^15^. Following the initial analysis with the nf-core/atacseq pipeline, further explorations into the data were carried out using customized bioinformatics tools including differential analysis and motif analysis. First, FastQC^16^ was used to produce quality metrics. Then adapter trimming was performed with Trim Galore, a comprehensive wrapper combining Cutadapt and FastQC, for the refinement of FastQ files^16,17^. We used default settings to identify and remove adapter sequences to streamline preprocessing steps without manual intervention.

Hisat2 was used to align sequencing data^18^. Trimmed ATAC-Seq reads were aligned against the *Hordeum vulgare* Morex V3 reference genome with the addition of *Bln1* (*Bln1* was not annotated in the original Morex V3 release)^19^. The output was transmitted into Samtools^20^, which was then converted into a compressed BAM file, filtering out secondary alignments, and leveraging multiple threads for efficiency. After alignment, reads were filtered using a combination of SAMtools, BEDTools, BAMTools, and Pysam (https://github.com/pysam-developers/pysam) to ensure high-quality alignments for downstream analysis^20–22^. SAMtools and BEDTools were used to remove reads marked as duplicates, non-primary alignments, unmapped reads, and those mapping to multiple genomic locations. BAMTools handled the exclusion of reads with excessive mismatches (more than four), soft-clipped alignments, large insert sizes (>2 kb), and provided additional quality control. Pysam was employed to filter out inter-chromosomal paired-end reads, those with improper FR orientation, and to remove both reads from any pair where a single read failed the criteria.

### Peak calling

Peak calling in ATAC-Seq is a step where regions of the genome with significantly higher read counts, indicative of open chromatin, are identified. These peaks are essential for understanding regulatory elements and gene expression patterns. MACS2 was applied to identify regions with narrow peaks or broad peaks^23^. Then, deepTools^24^ was used to convert filtered BAM files into BigWig format, employing Counts Per Million (CPM) as the default normalization method to prepare the data for such comparative analyses and further bioinformatics processing.

### Differential analysis

After obtaining the count matrix where each row is a consensus peak region and each column is a sample, we implemented a differential analysis to compute statistically significant differential peaks between pairwise comparisons through DESeq2^25^. A minimal pre-filtering step was performed to keep only rows that had at least 10 reads total. After differential tests were applied to each peak, p-values were calculated and adjusted to control false positives via multiple testing. This filtering threshold was automatically determined to maximize detection power (*i.e*., maximize the number of differential peaks detected) at a specified false discovery rate (FDR). Tables were generated to report the peak annotation, and statistics and p adjusted and fold change for each pairwise comparison.

### Correlation with RNA-Seq data

General metrics such as the distribution of the annotated peaks across different genomic elements (promoter, intron, exon, intergenic) were generated. For those peaks annotated in the promoter regions, a distribution of their distance to the transcription start site (TSS) was reported. Paired RNA-Seq datasets were compared to the ATAC-Seq differentially accessible peaks by using a p adjusted threshold of 0.001 for RNA-Seq and 0.005 for ATAC-Seq, considering that the expected number of false positives in the datasets was not larger than 1. Genomic regions of 1 Mb and 10 Mb were used to bin the genes and a hypergeometric test was performed to identify if a region was enriched in differentially accessible and expressed regions. Comparison between differentially expressed and differentially accessible regions was performed.

Lastly, an epistatic analysis to identify gene interactions between *Mla6* and *Bln1* was performed by using the method utilized by^6^. Data from the four progenitor and mutant genotypes was combined to calculate additive and epistatic regions classified into symmetric, masked, suppression, positive and negative. The resulting regions were compared to an equivalent analysis using RNA-Seq data by binning the genes in each category or by comparing epistatic regions with significant enrichment of each epistatic category.

## Data Records

Raw FASTQ and processed BAM ATAC-Seq reads, in addition to peak calling for the 64 samples, are located at the NCBI-Gene Expression Omnibus (GEO) under accession GSE307853 (https://www.ncbi.nlm.nih.gov/geo/query/acc.cgi?acc = GSE307853)^26^.

## Technical Validation

**Fig. 1:**
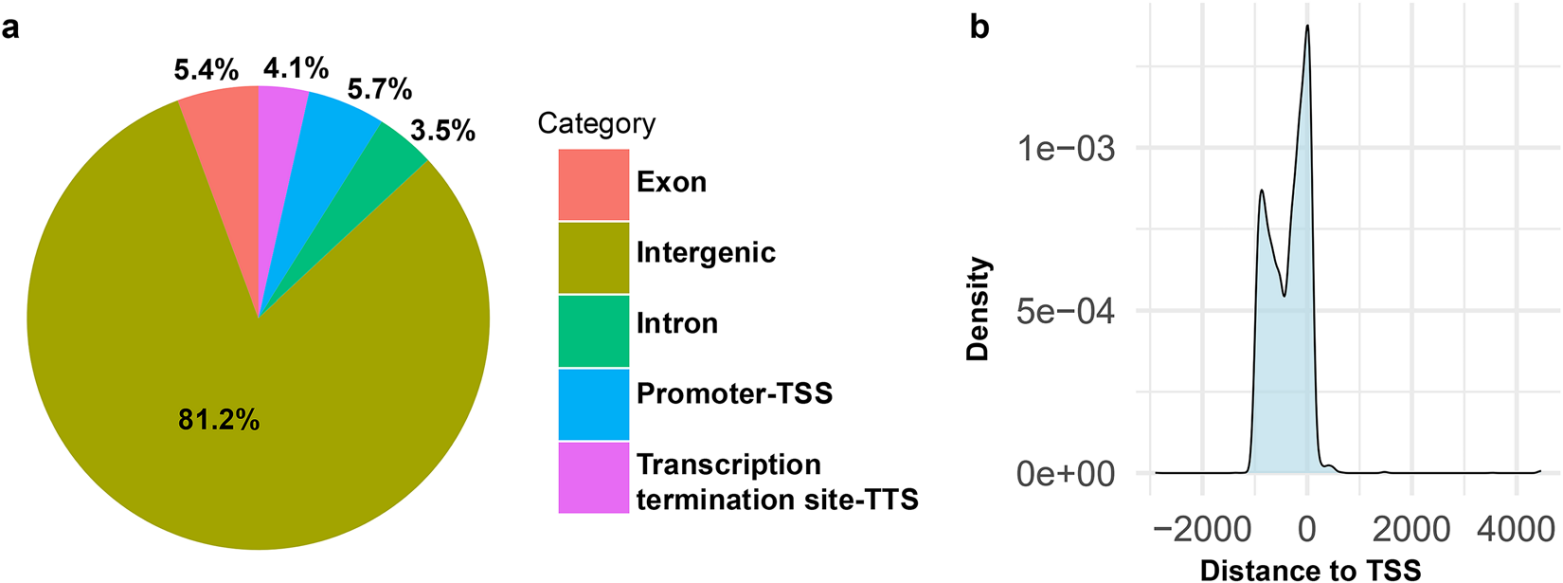
ATAC-Seq statistics. (**a**) Distribution of the peak annotations into genomic elements including exon, intergenic, intron, promoters-TSS and TTS (transcription termination site). (**b**) For the peaks annotated and Promoter-TSS, distribution of the peak distance to the transcription start site (TSS).

To validate the ATAC-Seq data we started by evaluating its contents given the annotation classification and the distribution of the distance of the ATAC-Seq peaks to the transcript start sites (TSS). Most of the peaks were found in intergenic regions (81.2%) followed by promoters with 5.7% and exonic regions with 5.4%. Intronic and transcription termination sites (TTS) had 4.1% and 3.5% respectively (Fig. 1a). Peaks classified in the promoters were located between 0 and 1000 bp upstream of the TSS (Fig. 1b). These results are congruent with typical ATAC-Seq datasets^27^ where most of the peaks are shown to be intergenic and the distance to the TSS is within 1000 bp upstream of this element.

**Fig. 2:**
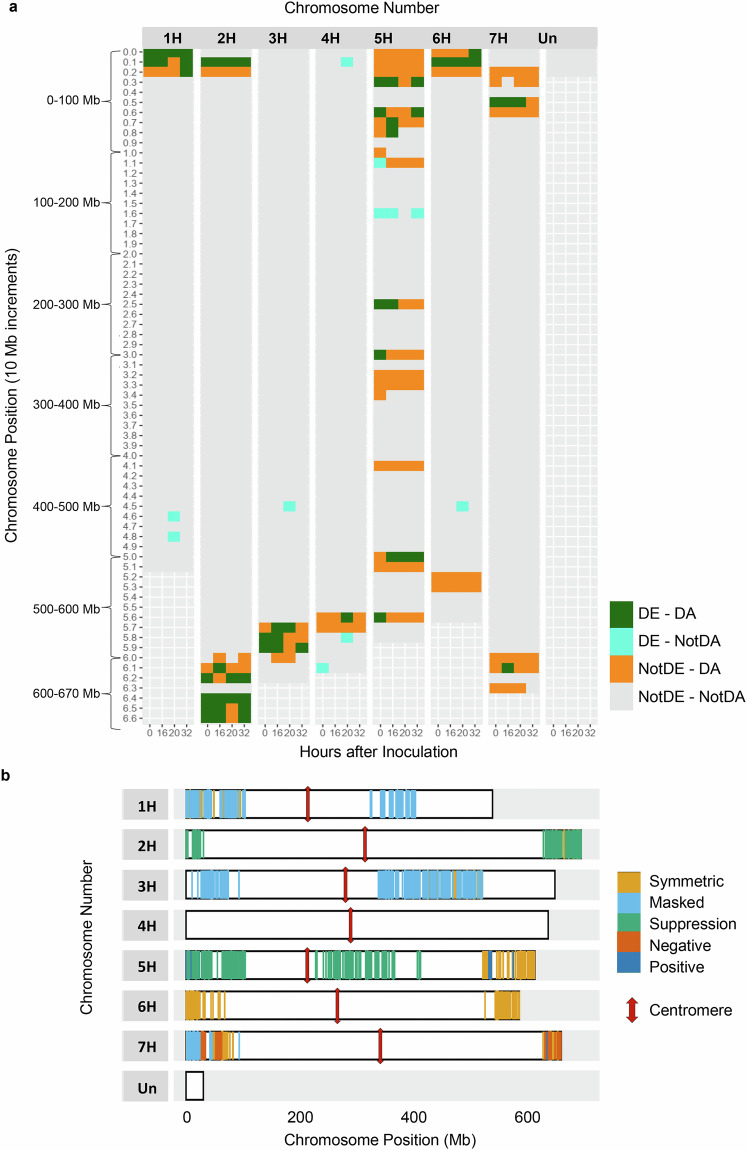
Correlation between ATAC-Seq and RNA-Seq data. (**a**) Correlation in the enrichment of DE and DA genes by genomic locations. Genes were binned in 1 Mb and 10 Mb windows and represented by chromosomal locations by separating each chromosome and bin. (**b**) Chromosome epistatic hotspots using ATAC-Seq. An epistasis model^6^ was applied to the ATAC-Seq data and epistatic chromatin hotspots were represented in five categories: symmetric, masked, suppression, positive and negative epistasis. Morex V3 centromere positions^29^ are indicated by double ended red arrows.

Next, the ATAC-Seq data was compared to RNA-Seq. Time-course ATAC-Seq datasets were compared to their corresponding RNA-Seq by classifying genes into differentially accessible (DA) differentially expressed (DE) and for each pairwise comparison of wild-type progenitor versus each of the other three mutants at each timepoint. Gene expression in barley can be regulated at large genomic distances from the gene promoter by distal *cis*-regulatory elements linked to their target genes through chromatin interactions^28^. Therefore, to test for correlation at the genomic scale, genes were binned into 1 Mb and 10 Mb groups and tested for enrichment of differentially accessible regions using a hypergeometric test. A similar test was performed for enrichment in differentially expressed genes, and the significant regions were compared. Figure 2a shows the results from this analysis, showing a strong correlation between differentially expressed and differentially distant accessible regions for both 1 and 10 Mb windows. Across time, a strong correlation between differential accessibility and expression was observed at positions 0 to 20 Mb on chromosomes 1H, 2H, 6H and 7H; 560 Mb to 590 Mb on chromosomes 3H, 4H, and 5H; between 600 Mb to 660 Mb on chromosomes 2H and 7H; and chromosome 5H from 0 to across multiple locations. Most of these genomic locations that were differentially accessible at early timepoints and differentially expressed later in the time course. These observations provided validation of the ATAC-Seq data as chromatin accessibility may enable transcription factor binding and activation.

An epistasis model to identify interactions between two genes in host immunity, the NLR-encoding *Mla6 and* the basal defense regulator *Bln1*, was also used to compare the two omic datasets. Sequenced genotypes comprised of CI 16151 wild-type progenitor (*Mla6, Bln1*), single mutants m18982 (*mla6, Bln1*) and m19089 (*Mla6, bln1*), and the double mutant m19028 (*mla6, bln1*) facilitated the evaluation of complex genetic models (*i.e*., genetic interactions) between *Mla6* and *Bln1*^6^. RNA-Seq analysis showed a genetic interaction between *Mla6* and *Bln1* with gene expression deviating from additive effects. Epistatic categories such as masked (where one gene’s effect hides the other), suppression (the inverse of masking) and symmetric (where mutants have similar effects) were observed in the dataset^6^. A similar analysis using the ATAC-Seq data showed congruent results, demonstrating that the epistatic relationship between *Mla6* and *Bln1* can be correlated to changes in chromatin accessibility. Figure 2b illustrates the chromosome hotspots enriched in each of the epistatic regions. Comparisons with the RNA-Seq epistatic hotspots^6^ indicated an overlap of 100% with ATAC-Seq in the chromosome 2H and 5H through symmetric epistasis; chromosome 1H, 3H and 7H are linked to masked epistasis and chromosome 2H and 5H are associated with suppression epistasis. Other hotspots found to be enriched with epistatic accessible regions include chromosomes 1H, 6H and 7H with symmetric epistasis and chromosomes 1H and 3H with masked epistasis. Notably, the ends of chromosome 2H and 7H positions 0 to 100 Mb are hotspots for multiple types of epistasis, indicating some overlap in genetic interaction at the chromatin level.

## Data Availability

Infection-time-course RNA-Seq datasets are available at NCBI-GEO under accession number GSE101304 (https://www.ncbi.nlm.nih.gov/geo/query/acc.cgi?acc=GSE101304).

## Code availability

Code used to perform these analyses is at Github repository https://github.com/vvelasqz/ATAC_seq under a MIT license and Zenodo 10.5281/zenodo.17072952.
